# Correction of pre-existing astigmatism with phacoemulsification using toric intraocular lens versus spherical intraocular lens and wave front guided surface ablation

**DOI:** 10.1186/s12886-022-02347-5

**Published:** 2022-03-12

**Authors:** Ahmed El-Shehawy, Ahmed El-Massry, Mohamed Sameh El-Shorbagy, Mohamed Atef, Moataz Sabry

**Affiliations:** 1 Department of Ophthalmology, Faculty of Medicine, Kafr-elsheikh University, Kafr-elsheikh, Egypt; 2 Department of Ophthalmology, Faculty of Medicine, Alexandria University, Champollion Street, Al Attarin, Alexandria, Egypt; 3 Department of Ophthalmology, Faculty of Medicine, Tanta University, Tanta, Egypt

**Keywords:** Astigmatism, Phacoemulsification, Toric intraocular lens, Spherical intraocular lens, Wave front guided surface ablation

## Abstract

**Background:**

This study aimed to evaluate toric intraocular lens to correct of pre-existing astigmatism at the time of phacoemulsification compared to using of spherical intraocular lens followed by wavefront guided surface ablation.

**Results:**

The patients were classified into three groups: Group A with 20 eyes of 19 patients having phacoemulsification with spherical intraocular lens only as a control group, group B with 20 eyes of 14 patients had phacoemulsification with toric intraocular lens and group C with 20 eyes of 16 patients had phacoemulsification with spherical intraocular lens and wavefront guided PRK three months later. Comparison pre-operative data for all groups showed no statistically significant difference regarding UCVA, BCVA, MRSE, and refractive astigmatism (*P*>0.05). Post operatively, there was a statistically significant difference for UCVA, BCVA, MRSE, and refractive astigmatism for group A compared to group B (*P*<0.05) and group A compared to group C but there was no statistically significant difference for group B compared to C regarding all these parameters (*P*>0.05).

**Conclusion:**

In this study, we found similar effects for both techniques in astigmatism corrected groups while both differed from the control group that was not corrected. Correcting preexisting astigmatism during cataract surgery should be in mind in every case to improve visual outcomes. Longer period of follow up are required to evaluate stability of these techniques and possibility of regression.

## Introduction

Cataract surgery has rapidly become one of the most widely performed surgeries in the world. However, preexisting corneal astigmatism often result in some degree of residual refractive error [1]. It is reported that around 70% of the general population with cataract has at least 1diopter (D) of astigmatism, and around 33% of cases undergoing cataract surgery can be treated of preexisting astigmatism [2]. These findings imply that, while planning a surgery, we should care about both the spherical and the astigmatic components to get post-operative outcomes close to emmetropia as far as possible. Moreover, the most critical factor in dealing with the astigmatism is to check the exact source, axis and magnitude of the astigmatism and to make the decision about the appropriate technique for each patient [3].

In the past, the aim of cataract surgery was just restorative to remove a cloudy lens usually with the help of glasses post-operatively [4]. A modern era of cataract surgery aims to obtain the most preferable refractive outcomes for the cases and decrease any need for other corrections [4]. Phacoemulsification could eliminate the lenticular astigmatism part. For the elimination of the corneal part, the surgeon should assess the meridian and amount of corneal astigmatism [5]. Another issue is the surgically induced astigmatism (SIA) that can be produced by cataract incisions. SIA should not be neglected, especially in case of low pre-existing astigmatism. That is why it can now be considered as “refractive cataract surgery” [5]. Refractive cataract surgery has been designed for a more aggressive aim; to enable cataract cases to regain better vision and to eliminate or reduce the need for more corrections greatly, including the reading glasses following surgery [5].

Zaldivar first described the concept of bioptics while performing LASIK in order to correct residual refractive error in eyes after receiving phakic IOLs. The results suggested this to be effective and predictable for correcting residual error in subjects with extreme preoperative myopia reaching up to -35.00D [6]. Moreover, Güell described adjustable refractive surgeries in which he performed LASIK for correcting residual ametropia following several sorts of intraocular surgeries which included implanting of both IOLs and phakic IOLs, corneal refractive techniques such as radial and arcuate keratotomy and PRK in addition to penetrating keratoplasties [7]. A study of 30 eyes with postsurgical ametropia found that 12 months after PRK, 93% of participants were within ±0.50 D of the target refraction [8]. Although both PRK and LASIK have been demonstrated to be safe and effective for the correction of residual refractive error after cataract surgery, in many cases, LASIK induces more severe and persistent damage to corneal sensation, corneal barrier function, and tear film stability than PRK [9]. The theoretical importance of this combined method include the maximization of the size of the optical zone and improving the predictability for the refractive outcome [10]. However, bioptics carries the risks of two procedures. From one side, it includes the risks for IOL surgery as bleeding and endophthalmitis and from the other side, there are risks for corneal refractive surgery such as infection, dry eye, irregular astigmatism, and keratectasia in addition to glare, halos, and ghost images [11]. Toric IOLs are used to correct astigmatism druing cataract surgery to reduce astigmatism post-operatively [12]. Shimizu et al firstly presented toric IOL in 1994. Postoperatively, about 20% of the IOLs rotated 30 degrees or more and almost 50% of IOLs rotated more than 10 degrees and have been used clinically since then [13]. Therefore, we aimed to evaluate toric IOL to correct pre-existing astigmatism at the time of phacoemulsification compared to using of spherical intraocular lens followed by wavefront guided surface ablation.

### Patients and methods

This retrospective comparative clinical study included 60 eyes of 49 patients who attended outpatient ophthalmology clinics in our University hospitals during 2017 and 2018 with visually significant cataracts indicated for phacoemulsification and co-existing regular astigmatism. We included patients diagnosed with visually significant cataracts and regular astigmatism between 1 to 4 D and completed follow-up. Exclusion criteria were the presence of any concurrent eye conditions that can affect the outcome of visual acuity as corneal scar, irregular astigmatism, glaucoma, chronic intraocular inflammations, lens sublaxation, posterior segment abnormalities, and previous refractive procedures. The patients were classified into three groups: Group A with 20 eyes of 19 patients who had phacoemulsification with spherical intraocular lens only as a control group, group B with 20 eyes of 14 patients had phacoemulsification with toric intraocular lens and group C with 20 eyes of 16 patients had phacoemulsification with spherical intraocular lens and wavefront guided PRK three months later.

Data included history taking for age, sex, any systemic or topical medications, and history of any previous ophthalmic disease or surgery. In addition, we collected the data of uncorrected visual acuity (UCVA) and best-corrected visual acuity (BCVA) using Snellen Chart, then visual acuity was converted to Log MAR for statistical analysis, manifest Refraction if possible according to density of cataract, intraocular pressure (IOP) measurement using Goldman applanation tonometer and sterile fluorescein strips, anterior segment slit lamp examination, tear film to exclude dry eye syndrome, cornea to exclude scars and any other clinically detectable abnormalities, and lens to grade opacification and exclude sublaxation. Moreover, fundus examination, biometry to Calculate IOL power using Zeiss IOL Master 500 Device, online toric IOL calculator (Tecnistoriccalc.com) to calculate desired IOL and main wound axes were done.

Informed consent for operations was obtained after discussing extensively with each patient about the benefits, risks, possible side effects of the procedure. The patients were prepared for the technique using topical antibiotics (Moxifloxacin hydrochloride 0.5%, Vigamox, Alcon, USA) 4 times daily 3 days before operation, topical NSAID and pupillary dilators (Mydrapid 1%, Alexandria, Egypt). The slit lamp was used to mark the principal meridians (0,180 and 270 axes) using a hand held ink marker in all patients. The technique was carried out under aseptic conditions in the operating room with an operating microscope. Local anesthesia in the form of topical and peribulbar blocks as lidocaine solution was used. Topical application of 10% povidone-iodine (Betadine, Nile/Mundi) for periocular area, lids and eyelashes was done before any procedure. The patient was draped completely and an eyelid speculum was used. Drops of 5% povidone-iodine (Betadine, Nile/Mundi) were instilled into the conjunctival sac for 2 to 3 minutes and then washed by sterile normal saline. In group A and C, the site of main wound was marked on the steep axis defined by preoperative biometry depending on the previous main meridians marked on the slit lamp preoperatively using a holed Mendez ring marker. In group B, the desired axis of toric IOL as well as main incision were marked depending on planned map and the previous main meridians marked on the slit lamp preoperatively using a holed Mendez ring marker. Procedure started with making a side port entry and injecting viscoelastic in the anterior chamber. It was made in clear cornea with 20 G MVR blade. The side port should measure about 1 mm and run parallel to iris plane. After supporting the globe by placing a toothed forceps outside limbus opposite to the site of making side port and AC was entered with MVR blade. on the planned previously marked axis in group B or on the steepest previously marked axis in groups (A and C) main wound was done using 2.4 mm keratome that was pushed into the depth of the wound and angled forward into the layers of the cornea for about 1.5mm. Direction of keratome was forward and upward following the curve of cornea. Then the direction of keratome was changed downward to cut the Descemet's membrane and penetrate into the A.C. Standard phacoemulsification was performed. In group B, A toric IOL (Tecnis J and J Company USA) is implanted on irrigating fluids and rotated to match the marks on it with the marks previously done on the limbus. In group A and C, A foldable acrylic IOL was implanted inside the bag. Meticulous removal of any viscoelastic materials and hydration of the main incision and side port by using balanced saline solution (BSS) and recheck toric IOL orientation after removal of eye speculum. The surgeons who performed operations and the authors of this study were the same.

Postoperative treatment included topical antibiotic eyedrops (Moxifloxacin hydrochloride 0.5%, Vigamox, Alcon, USA) five times daily and topical corticosteroids eyedrops (Prednisoline acetate, Econopred plus 1%, Alcon, USA) every two hours for the first day then tapered over one month. The patients were examined for follow up at one day under dilatation in group B to check toric IOL alignment then three days, one week, one month and three months after the operation.

For group C, Topical antibiotic (Vigamox) was applied 2 days before the procedure and topical anesthesia (Benox) was added frequently at the start then periocular area was sterilized by topical application of 10% povidone-iodine (Betadine, Nile/Mundi) for periocular area, lids and eyelashes before any procedure. Then, draped completely and an eyelid speculum was used to stabilize the eyelids. Mechanical removal of central 9 mm of epithelium using a hockey knife guided by 9 mm ring print was performed. Activation of pupil and iris registered tracking system was done. After centration was done, Excimer laser photoablation using wave front custom analysis (Star S4 IR Excimer laser, Amo internationals, USA). MMC 0.05% was applied for 20- 30 seconds. Copious irrigation used for at least 30 cmm of BSS. A bandage soft contact lens was applied at the end of procedure Postoperative topical antibiotic eyedrops (Moxifloxacin hydrochloride 0.5%, Vigamox, Alcon, USA) five times daily, topical corticosteroids eyedrops (Prednisoline acetate, Econopred plus 1%, Alcon, USA) every two hours for the first day then tapered over one month, topical cycloplegia three times for five days to decrease postoperative pain and any preservative free lubricants for six weeks. The patients were examined for follow up till three months after the operation.

The sample size was calculated as a minimum of 15 eyes in each group to get a power of 0.8 and an alpha error of 0.05 which goes in line with several studies comparing toric IOLs to different treatment options with sample size of 20 or less for each group [14–16]. Kolmogrov-semornov and Shapiro Wilk tests were used to assess the normality of the numerical data. Statistical description and analysis of the present study was conducted using the mean, standard deviation, and range for descriptive statistics, T-test, Mann-Whitney test and Wilcoxon Signed Ranks test for inferential statistics by SPSS V.18 Software (SPSS Inc., Chicago, IL).

## Results

In this study, the range of age in group A was from 45 to 66 years with a mean value of 55.20±6.10 years. In group B, it was found that the range of age was from 47 to 70 years with a mean value of 53.20±5.60 years. In group C, it was found that the range of age was from 42 to 60 years with a mean value of 50.60±5.10 years. There was no statistically significant difference in age between all groups (*p*=0.06). As regard the sex in the three groups, it was found that group A included 19 patients (10 females and 9 males), group B included 14 patients (9 females and 5 males) and group C included 16 patients (9 females and 7 males). Regarding laterality, it was found that group A included (13 right and 7 left), group B included (8 right and 12 left) and group C included (10 right and 10 left) with no significant difference among all groups (*p*=0.28). According to this study, it was found that the range of biometry cylinder in group A was 1.52 to 3.3 D with a mean value of 2.3±0.47 D. In group B, it was found that the range of biometry cylinder was 1.1 to 4 D with a mean value of 2.95±0.8 D. In group C, it was found that the range of biometry cylinder was 1.55 to 3.64 D with a mean value of 2.74±0.61 D. There was no statistically significant difference in biometry cylinder between all groups (*p*=0.38).

Comparison pre-operative data for all groups showed no statistically significant difference regarding UCVA, BCVA, MRSE, and refractive astigmatism (*P*>0.05) as shown in Table 1. Post operatively, there was a statistically significant difference for UCVA, BCVA, MRSE, and refractive astigmatism for group A compared to group B (*P*<0.05) and group A compared to group C (*P*<0.05) but there was no statistically significant difference for group B compared to C regarding all these parameters B (*P*>0.05, Table 2).

**Table 1:** Preoperative data of group A, B and C regarding UCVA, BCVA, MRSE, and refractive astigmatism

Preoperative	Group A	Group B	Group C	***P***- value
UCVA	1.02± 0.48(0.4:2)	1.09±0.52(0.04:2.08)	1±0.42(0.52:2)	0.91
BCVA	0.46±0.24(0.22:1)	0.36±0.12(0.22:0.7)	0.43±0.15(0.22:0.7)	0.11
MRSE	-1.3±3.7(-9:5)	-5.3± 5(-20.5:0.25)	-2.8±3.5(-8:6)	0.15
Refractive Astigmatism	2.7±0.84 (1.25:4)	3.4±1.1 (1.5:5)	3±0.8 (1.75:4.5)	0.3

**Table 2:** Postoperative data of patients in groups A, B and C regarding UCVA, BCVA, MRSE, and refractive astigmatism

Postoperative	Group A	Group B	Group C	***P***- value (A vs B)	***P***- value (A vs C)	***P***- value (B vs C)
UCVA	0.36± 0.13 (0.097:0.52)	0.14±0.1 (0:0.4)	0.114±0.056 (0.046:0.22)	<0.001	<0.0001	0.82
BCVA	0.185±0.1 (0:0.3)	0.09±0.1 (0:0.4)	0.06±0.04 (0:0.16)	0.003	0.00006	0.62
MRSE	-0.76±0.64(-1.75:1.25)	-0.36± 0.4(-1:0.25)	-0.18±0.35(-1:0.25)	0.004	0.0002	0.11
Refractive Astigmatism	2.15±0.6 (1:3.5)	0.53±0.32 (0:1.25)	0.4±0.15(0.25:0.75)	<0.00001	<0.00001	0.09

As regards the changes in each group, there was a significant improvement in UCVA by log MAR in group A as it improved from 1.02± 0.48(0.4:2) preoperatively to 0.36± 0.13(0.097:0.52) postoperatively (*p* <0.0001). Moreover, there was a significant improvement in BCVA by log MAR in group A as it improved from 0.46±0.24 (0.22:1) preoperatively to 0.185±0.1 (0:0.3) postoperatively (*p* <0.0001). There was a reduction in MRSE in group A as it changed from - 1.3±3.7 (-9:5) preoperatively to -0.76±0.64 (-1.75:1.25) postoperatively but it was not statistically significant (*p*=0.55). There was a slight significant reduction in refractive astigmatism in group A as it changed from 2.7±0.84 (1.25:4) preoperatively to2.15±0.6 (1:3.5) postoperatively (*p*=0.031). Regarding group B, There was a significant improvement in UCVA by log MAR in group B as it improved from 1.09± 0.52(0.04:2.08) preoperatively to 0.14± 0.1(0:0.4) postoperatively (*p* <0.001). Moreover, there was a significant improvement in BCVA by log MAR in group B as it improved from 0.36± 0.12(0.22:0.7) preoperatively to 0.09± 0.1(0:0.4) postoperatively (*p* <0.001). There was a significant reduction in MRSE in group B as it changed from -5.3± 5(-20.5:0.25) preoperatively to -0.36± 0.4(-1:0.25) postoperatively (*p* <0.001). There was a marked significant reduction in refractive astigmatism in group B as it changed from 3.4± 1.1(1.5:5) preoperatively to 0.53± 0.32(0:1.25) postoperatively (*p* <0.001). Regarding the changes in group C, there was a significant improvement in UCVA by log MAR in group C as it improved from 1±0.42 (0.52:2) preoperatively to 0.114±0.056 (0.046:0.22) postoperatively (*p* <0.001). Also there was a significant improvement in BCVA by log MAR in group C as it improved from 0.43±0.15 (0.22:0.7) preoperatively to 0.06±0.04 (0:0.16) postoperatively (*p* <0.001). There was a significant reduction in MRSE in group C as it changed from -2.8±3.5 (-8:6) preoperatively to -0.18±0.35 (-1:0.25) postoperatively (0.008). There was a marked significant reduction in refractive astigmatism in group C as it changed from 3±0.8 (1.75:4.5) preoperatively to 0.4±0.15 (0.25:0.75) postoperatively (*p* <0.001). Vector analysis of all groups is shown in Table 3. For Absolute angle of error of all groups, there was a significant difference between A and B, A and C but there was no significant difference for B and C as shown in Table 4.

**Table 3:** Vector Analysis in all groups using Alpins data analysis

Parameter		Group A	Group B	Group C
Target Induced Astigmatism (TIA)		2.7 ± 0.84 (1.25:4)	3.41 ± 1.13 (1.5:5)	3.03 ± 0.79 (1.75:4.5)
	Mean vector	1.45 axis 84	0.94 axis 174	1.21 axis 179
Surgical Induced Astigmatism (SIA)		1.66±1.46 (0.3:5.5)	3.47±1.14 (1.5:5.47)	2.9±0.88 (1.5:4.6)
	Mean vector	0.88 axis 76	0.93 axis 177	0.84 axis 0
Difference Vector (DV)		2.15±0.62 (1:3.5)	0.53±0.32 (0:1.25)	0.4±0.15 (0.25:0.75)
	Mean vector	0.65 axis 95	0.093 axis 133	0.37 axis 177
Magnitude of Error (ME)	+ =overcorrection - = undercorrection	-1±1.4 (-2.8:1.79)	0.06±0.35 (-0.74:0.55)	-0.13±0.34 (-0.73:0.48)
Correction Index (CI)	1 means ideal	0.47 (0.17:1.6)	1.02 (0.82:1.32)	0.95 (0.67:1.23)
Angle of error (AE) by degrees		-22 ± 49.5 (-156:29.8)	3 ± 3.5 (-3.1:7.22)	0.5 ±2.6 (-2.7:7.20)
Absolute AE by degrees		34 ±42 (2.3:156)	3.14 ± 2.53 (0:7.22)	1.86 ±1.78 (0:7.20)
Torque Effect (TE)		-0.22 ± 1.15 (-2.82:2.25)	0.26 ± 0.45 (-0.43:1.25)	0.02 ± 0.24 (-0.3:0.5)
Flattening Effect (FE)		1.3 ±1.36 (-0.12:4.77)	3.44 ± 1.11 (1.5:5.46)	2.89 ± 0.83 (1.5:4.59)
Index Of Success (IOS)		0.8±0.13 (0.53:1.14)	0.16± 0.1 (0:0.43)	0.13± 0.07 (0.07:0.33)
Percentage of success		19% (14:46.6%)	83.9 % (57:100%)	85.9% (66.7:92.8%)

**Table 4:** Absolute angle of error of all groups

Groups	*P* –Value of absolute angle of error
Groups A& B	<0.0001*
Groups A&C	<0.0001*
Groups B&C	0.133

As regard complications in all groups: no recorded cases of wound leakage, IOL decentration and endophthalmitis. In group A, there were 5 eyes (25%) that developed corneal edema immediately postoperatively that was reversible with medical treatments,2 eyes (10%) that developed postoperative uveitis that was reversible with medical treatments and 3 eyes (15%) that developed posterior capsular opacification (PCO). In group B, there were 6 eyes (30%) that developed corneal edema that was reversible with medical treatment, 2 eyes (10%) that developed PCO and only one eye (5%) developed postoperative AC reactions immediately postoperative that was reversible with medical treatments and 4 eyes (20%) that developed significant rotation of IOL and were in need of re alignment. In group C, there were 3 eyes (30%) that developed corneal edema that was reversible with medical treatment, only one eye (5%) developed postoperative AC reactions immediately postoperative that was reversible with medical treatments and 5 eyes (25%) that developed dry eye and needed long lasting lubricants.

## Discussion

We compared toric IOL implantation (one step surgery) and customized photorefractive keratectomy post phacoemulsification (two steps surgery) and found comparable results for both techniques in astigmatism corrected groups while both differed from the control group that was not corrected. Although PRK may lead to better results as we can measure astigmatism more precisely after the primary surgery, being the two step surgery, increase time to complete recovery, costs, need for high tech diagnostic (wavefront analysis) are considered major disadvantages. Moreover, substantially same effect of primary toric IOL and Wavefront guided PRK could be a strong factor in favor of topic IOL use that is simpler, faster and overall cheaper one step surgery. Ritu Nagpal et al reported that 86.6% (26/30) of toric IOL eyes had residual refractive cylinder < 0.5 D while in PRK eyes this value was 96.6% (29/30). None of the patients had residual cylinder > 0.75 D. The spherical equivalent value was < 0.5 D in 86.6% (26/30) of toric IOL eyes and 93.3% (28/30) of PRK eyes [17]. Also UDVA ≥ 20/20 (6/6) was seen in 53.3% (16/30) of toric IOL eyes and 60% (18/30) of PRK eyes [17]. In our study, we found that 75% (15/20) of toric IOL eyes had residual refractive cylinder < 0.5 D while in PRK eyes this value was 95% (19/20). The spherical equivalent value was < 0.5 D in 80% (16/20) of toric IOL eyes and 85% (17/20) of PRK eyes. Also UDVA ≥ 20/20 (6/6) was seen in 55% (11/20) of toric IOL eyes and 60% (12/20) of PRK eyes. Toric IOL has been shown to be a simple and effective method to correct astigmatism during cataract surgery. Using toric IOLs is a desirable technique to decrease pre-existing astigmatism with cataract surgery in eyes different degrees of astigmatism. They also sound to have potential pros compared to arcuate keratotomy or corneal incisions through being an easy to perform and stable method with high and advanced technology. Holland et al study showed that in around 60% of cases with toric IOLs achieved spectacle independency compared to 36% of cases having control IOLs [18]. Lane et al also showed cases from the study of Holland et al fellow-eye implantation through the same IOL (toric or control IOL), with an opportunity to examine bilaterally for spectacle independency and 97% of the participants had spectacle independency with toric IOLs for distance vision compared to half of the participants in the control group [19]. Visser et al toric IOL group had better UDVA with refractive astigmatism lower than control IOL group [11]. Holland et al showed that UDVA was 20/25 or even better in around 60% to 80% of subjects having toric IOLs. For refractive astigmatism, there was a correction index of 1.20 and magnitude of error of +0.38 D with general overcorrection for astigmatism through Acrysof toric IOLs [20]. Goggin et al also showed this overcorrection and is correlated with the underestimation of the power for IOL cylinder at the the manufacturer corneal plane [21]. In our study, there was improvement in UCVA, BCVA, MRSE, and reduction of refractive astigmatism as mean astigmatism changed from 3.4 ± 1.1D (1.5:5) preoperatively to 0.53 ± 0.32D (0:1.25) postoperatively. The main disadvantages of toric IOL are precise calculation of IOL and exact cylinder and marking orientation also the rotation of IOL immediately postoperatively that may be enough to abolish its action and be in need for repositioning . Moreover, they are noted to be avoided in ectatic corneal disorders because the outcomes may be unpredictable.

In our study, there were 6 eyes (30%) that developed corneal edema that was reversible with medical treatment, 2 eyes (10%) that developed PCO and only one eye (5%) developed postoperative AC reactions immediately postoperative that was reversible with medical treatments and 4 eyes (20%) that developed significant rotation of IOL and were in need of re alignment In customized photorefractive keratectomy, Excimer laser is used to correct residual errors guided by wavefront aberrations analysis done before the procedure Excimer procedures may be predictable in correcting refractive errors of lower amplitude showed in most patients. By using wavefront guided ablation, high order aberrations as well as possibility of decentration were eliminated. Sáles et al provided a review for several strategies to handle the refractive errors following cataract including arcuate keratotomy, LASIK, PRK and other intraocular approaches as IOL exchange, piggyback IOLs, and light-adjustable IOLs. The laser vision correction was more effective with predictable outcomes compared to intraocular surgeries with their potential risks [22]. A retrospective clinical study included patients with an unacceptable final refractive error following phacoemulsification and compared intraocular approach with IOL exchange, piggyback lens and LASIK. Despite that all of the procedures were effective, the LASIK showed the best outcomes [23]. A study by Jin et al used both LASIK and lens-based surgery to correctresidual refractive error after cataract surgery and found that both procedures can be regarded as effective and predictable procedures [24]. On a theoretical basis, IOL exchange and piggyback IOLs may be better compared to surface treatments. However, the intraocular procedures have their potential risks for severe complications including endophthalmitis and capsular rupture. Therefore, the excimer laser ablation is more perfered to avoid those complications [25]. Aragona et al reported that PRK can be regarded safe and effective to correct residual refractive errors after cataract with stable long term results [26]. In our study, there was improvement in UCVA, BCVA, MRSE, and reduction of refractive astigmatism as mean astigmatism changed from 3 ± 0.8D (1.75:4.5) preoperatively to 0.4 ± 0.15 D (0.25:0.75) postoperatively. Disadvantages of this method include postoperative pain, the possibility of haze formation and dry eye syndrome.

In our study, there were 3 eyes (30%) that developed corneal edema that was reversible with medical treatment, only one eye (5%) developed postoperative AC reactions immediately postoperative that was reversible with medical treatments and 5 eyes (25%) that developed dry eye and needed long lasting lubricants. In our study, there were no recorded cases of haze formation and endophthalmitis. Toric IOL implantation is a useful and significant method to correct corneal astigmatism of 1.5 D or more with cataract surgery in one stage procedure. To achieve good results, we should have very accurate k readings or even Pentacam. Another important factor is the preoperative IOL calculation and precise markings. Also, meticulous IOL examination in the early postoperative periods for accurate centration and alignment as rotation is a common postoperative complication which needs immediate interference and realignment especially if significant and more that 10 degrees off the desired axis. In order to decrease incidence of rotation, we recommend implantation of IOL on irrigating fluids not viscoelastic materials, meticulous removal of any viscoelastic materials and reexamine IOL alignment after removal of eye speculum. We found similar effects for both techniques in astigmatism corrected groups while both differed from the control group that was not corrected. Correcting preexisting astigmatism during cataract surgery should be in mind in every case to improve visual outcomes. Longer period of follow up are required to evaluate stability of these techniques and possibility of regression.

## Data Availability

The data used to support the findings of this study are available from the corresponding author upon request.
